# Supplementary material for: The effect of different materials under the fencing piste on impact shock of the tibia during the fencing lunge on a concrete surface
Source: PLoS One. 2025 May 15;20(5):e0323557. doi: 10.1371/journal.pone.0323557 (PMC12080814; doi:10.1371/journal.pone.0323557)
Supplement: Attachment — Submitted filename: Reviewer Responses v2.docx [file pone.0323557.s003.docx]

Response to Reviewers

Thank you for the comments to our paper.

Editorial Comments and Responses:

Please review your reference list to ensure that it is complete and correct. If you have cited papers that have been retracted, please include the rationale for doing so in the manuscript text, or remove these references and replace them with relevant current references. Any changes to the reference list should be mentioned in the rebuttal letter that accompanies your revised manuscript. If you need to cite a retracted article, indicate the article’s retracted status in the References list and also include a citation and full reference for the retraction notice.

**Response**: We have not made any amendments and the references seem to be correct.

Reviewer comments:

Reviewer #2:

**Reviewer**: Adding a figure displaying the pattern (mean and SD as a function of time) of the recorded accelerations (not only the peaks, as in Figure 2) could help the reader and facilitate the replication of the study in future research.

**Response**: We have discussed this as a team but we do not feel it appropriate to add this figure. When we wrote the ethics application we planned to analyse the peak impact accelerations as this is what we are most interested in. We therefore do not want to deviate away from our pre planned analyses. Secondly, this is the method we have previously used and published (Sinclair et al., 2010 and Greenhalgh et al., 2013). Thirdly, to do this figure would mean rerunning all the analyses and using SPM method for statistics. Also, we do not have the exact start and end times for the lunges as we have done this in the field which means we can’t accurately time normalise it. Therefore, this wouldn’t provide any additional clarity to the reader. We feel our current figure is the most appropriate to best represent the data.

**Reviewer**: Even if an author is present in the photo, it is advisable to blur their face to ensure anonymity and respect privacy.

**Response**: We have now blurred the face of the fencer.
